# Respiratory function declines in children with asthma associated with chemical species of fine particulate matter (PM_2.5_) in Nagasaki, Japan

**DOI:** 10.1186/s12940-021-00796-x

**Published:** 2021-10-21

**Authors:** Yoonhee Kim, Eun Ha Park, Chris Fook Sheng Ng, Yeonseung Chung, Kunio Hashimoto, Kasumi Tashiro, Hideki Hasunuma, Masataka Doi, Kei Tamura, Hiroyuki Moriuchi, Yuji Nishiwaki, Hwajin Kim, Seung-Muk Yi, Ho Kim, Masahiro Hashizume

**Affiliations:** 1 Department of Global Environmental Health, Graduate School of Medicine, The University of Tokyo, Tokyo, Japan; 2 Institute of Health and Environment, Seoul National University, Seoul, South Korea; 3 School of Tropical Medicine and Global Health, Nagasaki University, Nagasaki, Japan; 4 Department of Mathematical Sciences, Korea Advanced Institute of Science and Technology, Daejeon, South Korea; 5 Department of Paediatrics, Graduate School of Biomedical Sciences, Nagasaki University, Nagasaki, Japan; 6 Department of Paediatrics, Isahaya General Hospital, Nagasaki, Japan; 7 Department of Public Health, Hyogo College of Medicine, Nishinomiya, Hyogo Japan; 8 Kenhoku Healthcare Office, Nagasaki Prefectural Government, Nagasaki, Japan; 9 Pharmaceutical Administration Office, Nagasaki Prefectural Government, Nagasaki, Japan; 10 Department of Environmental and Occupational Health, School of Medicine, Toho University, Tokyo, Japan; 11 Graduate School of Public Health, Seoul National University, Seoul, South Korea; 12 Department of Global Health Policy, Graduate School of Medicine, The University of Tokyo, 7-3-1 Hongo, Bunkyo-ku, Tokyo, 113-0033 Japan

**Keywords:** Particulate matter, Chemical composition, Asthma, Lung function, Children

## Abstract

**Background:**

The differential effects of PM_2.5_ fractions on children’s lung function remain inconclusive. This study aimed to examine whether lung function in asthmatic children was associated with increased PM_2.5_ fractions in urban areas in Nagasaki prefecture, Japan, where the air pollution level is relatively low but influenced by transboundary air pollution.

**Methods:**

We conducted a multiyear panel study of 73 asthmatic children (boys, 60.3%; mean age, 8.2 years) spanning spring 2014–2016 in two cities. We collected self-measured peak expiratory flow (PEF) twice a day and daily time-series data for PM_2.5_ total mass and its chemical species. We fitted a linear mixed effects model to examine short-term associations between PEF and PM_2.5_, adjusting for individual and time-varying confounders. A generalized linear mixed effects model was also used to estimate the association for worsening asthma defined by severe PEF decline. Back-trajectory and cluster analyses were used to investigate the long-range transboundary PM_2.5_ in the study areas.

**Results:**

We found that morning PEFs were adversely associated with higher levels of sulfate (− 1.61 L/min; 95% CI: − 3.07, − 0.15) in Nagasaki city and organic carbon (OC) (− 1.02 L/min; 95% CI: − 1.94, − 0.09) in Isahaya city, per interquartile range (IQR) increase at lag1. In addition, we observed consistent findings for worsening asthma, with higher odds of severe PEF decline in the morning for sulfate (odds ratio (OR) = 2.31; 95% CI: 1.12, 4.77) and ammonium (OR = 1.73; 95% CI: 1.06, 2.84) in Nagasaki city and OC (OR = 1.51; 95% CI: 1.06, 2.15) in Isahaya city, per IQR increase at lag1. The significant chemical species were higher on days that could be largely attributed to the path of Northeast China origin (for sulfate and ammonium) or both the same path and local sources (for OC) than by other clusters.

**Conclusions:**

This study provides evidence of the differential effects of PM_2.5_ fractions on lung function among asthmatic children in urban areas, where the Japanese national standards of air quality have been nearly met. Continuous efforts to promote mitigation actions and public awareness of hazardous transboundary air pollution are needed to protect susceptible children with asthma.

**Supplementary Information:**

The online version contains supplementary material available at 10.1186/s12940-021-00796-x.

## Background

Considerable evidence suggests that short-term exposure to ambient particulate matter (PM) is one of the leading factors that exacerbates pre-existing asthma by inducing airway inflammation [1, 2]. Globally, asthma exacerbation attributable to short-term exposure to PM_2.5_, meaning particulate matter ≤2.5 μm in aerodynamic diameter, was estimated at 5–10 million asthma emergency room visits in 2015, accounting for 4–9% of annual global visits [3].

Since PM is a complex mixture of aerosols, many epidemiological studies have attempted to determine which components of the particle mixture are more harmful to health. For example, some elements of PM_2.5_, such as elemental carbon (EC) and organic carbon (OC) from coal combustion and vehicle emissions, were associated with increased risks of mortality or hospital admissions for cardiorespiratory diseases [4–6]. Evidence for asthma exacerbation associated with specific components of PM_2.5_ has also accumulated [7–9], and growing attention has focused on the role of traffic-related air pollution (TRAP) in asthma [1]. Given that distributions of chemical components of PM mixtures vary by area and season depending on different characteristics of emission sources [10], understanding the differential toxicity of PM_2.5_, which could also differ by area, is necessary to effectively regulate the sources of toxic elements for a given region.

Nagasaki, located in the westernmost part of the main islands of Japan, is in a unique position where the concentration of PM_2.5_ has been relatively low. However, the prefecture witnesses occasional rises in PM_2.5_ concentrations because of high levels of transboundary air pollution, such as from Asian Dust (AD) events [11], particularly in the spring when prevailing wind and subsequent downwind of AD (from northwest toward southeast) has occurred more frequently in East Asia. A previous study examined the chemical components of PM_2.5_ across neighboring Northeast Asian regions, including Beijing, Seoul, and Nagasaki [12], in which the characteristics of PM_2.5_ in Nagasaki city were somewhat different from those in Beijing and Seoul. This suggests that there was a lack of direct contribution from mobile and local industrial sources in Nagasaki city, but relatively stronger signals of long-range transport of air pollutants.

In recent decades, there has been growing evidence that cleaner air improves children’s lung function [13], based on a counterfactual condition observed in places with improved air quality, mostly in developed countries [14]. However, the concern remains whether the current standards of air quality are considered adequate because no threshold exists as a safe level of air pollution. The risks of mortality and morbidity were still observed in areas with low levels of air pollution [15, 16].

In this study, we aimed to examine whether lung function changes in children with asthma were associated with increased PM_2.5_ total mass and its chemical species in urban areas in Nagasaki prefecture, Japan, where the air pollution level is relatively low. We also performed back-trajectory and cluster analyses for PM_2.5_ to investigate multiple paths of transboundary air pollution in our study areas and calculated the contributions of each path to PM_2.5_.

## Methods

### Study design

We conducted a multi-year panel study spanning spring 2014–2016 in two neighboring cities of Nagasaki prefecture in Japan (Nagasaki city for February–June and Isahaya city for February–May; approximately 20 km apart, as shown in Figure S1). Nagasaki city is the capital city of Nagasaki prefecture with a population of 429,508 in 2015, which has traditionally been a large seaport with the condition widely open to the East China Sea. Isahaya city is the third populous city in Nagasaki prefecture with a population of 138,078 in 2015, located inside the prefecture.

In this study, a total of 73 children with asthma were enrolled and diagnosed with bronchial asthma by a medical doctor at either Nagasaki University Hospital or Isahaya General Hospital. In February every year during the study, we conducted a baseline survey for all subjects and collected basic information such as sex, age, body height and weight, and micro-environmental conditions such as secondhand smoking status (yes or no) and frequency of air cleaner use at home (every day, sometimes, or never). We also collected daily lung function measurements for children with asthma during the study period. Specifically, parents of the subjects were requested to make diary entries every day for their children with respect to self-measured peak expiratory flow (PEF) (described below) in conjunction with observed clinical symptoms, medication use, and hours spent engaged in outdoor activities.

### Daily PEF measurements and worsening asthma

We requested the self-measured PEF (L/min) twice a day, once in the morning and evening, in which a record was chosen as the highest PEF among three measurements. A Wright PEF meter (Clement Clarke International, London, UK) was used for the measurement obtained before the subject inhaled either corticosteroids or β2–agonists. All subjects and their parents were trained at the beginning of the study and they practiced taking the measurements for a month in February every year. This early period of training data was excluded from statistical analyses.

To define episodes of worsening asthma, we first calculated the percent change in the daily PEF variations from the personal best monthly PEF. We then converted the daily percent changes to a binary indicator for the episodes, based on two cut-off values if the percent reductions were greater than 15% and 20%, respectively, from the personal best monthly PEF [17, 18]. To avoid a potential bias for the episode definition possibly generated by the repeated episodes among neighboring days, which might not be triggered by the PM_2.5_ exposures, we included the first episode within a rolling 1-week window.

### PM_2.5_ mass and chemical composition

We collected daily time-series data of PM_2.5_ total mass and its chemical species measured at a single sampling site for each city. In Nagasaki city, we installed a sampling site on the rooftop of the Institute of Tropical Medicine of Nagasaki University (approximately 12 m above ground), located next to the Nagasaki University Hospital. A 24-h PM_2.5_ sample was measured at noon every three days but measured every day when an AD event was forecast by the Japanese Meteorological Agency (JMA). In total, the samples were available for 205 days during the study period. For Isahaya city, we obtained data from the Nagasaki Prefectural Institute of Environment and Public Health. The samples, measured on the ground level at noon every day, were available for 183 days. Detailed information about the number of sampling days by month and year is provided in the Supplementary Materials, Table S1.

Since we collected PM_2.5_ data from two different sites operated by the different institutes, we attempted to make the study setting comparable as much as possible. Thus, we selected PM_2.5_ mass and five chemical species for our statistical analyses, which were measured in both cities and accounted for a large fraction of PM_2.5_ mass (41.7% in Nagasaki city and 69.9% in Isahaya city), including carbonaceous constituents (EC and OC) and water-soluble inorganic ions (SO_4_^2−^, NO_3_^−^, and NH_4_^+^). However, we acknowledge that the methods of PM_2.5_ sampling and analysis between the two sites were not strictly identical, although similar filters with the same material were used (i.e., polytetrafluoroethylene [PTFE] filters for PM_2.5_ mass and Quartz filters for carbonaceous species). To avoid any misleading results based on the difference between the sites, we performed all analyses, including health risk assessment and back-trajectory and cluster analysis described below, separately for Nagasaki city and Isahaya city. Details about the PM_2.5_ sampling and analysis methods such as instruments, sampling flow, type of filters, methods of analyzing filters, and laboratory instrumentation and techniques for each site are described in the Supplementary Materials.

To deal with a few samples below the detection limit (DL), we calculated year-specific (i.e., spring) minimum values as the DL and substituted a DL/2 for the cases below the DL. The imputation was implemented on eight days in total (i.e., one day for EC and nitrate, respectively, and two days for sulfate and ammonium, respectively, in Nagasaki city; two days for nitrate in Isahaya city). In addition, we calculated year-specific averages to be used for another imputation on missing values of the five elements (i.e., the same seven days for the five elements in Isahaya city) only if the PM_2.5_ mass was available. No missing values were found in the city of Nagasaki.

### Meteorological data

We collected daily mean temperature (°C) and relative humidity (%) data from a single monitoring station in Nagasaki city through the JMA website (https://www.data.jma.go.jp/gmd/risk/obsdl). The same data were used for the two cities because no observation data were available in Isahaya city.

### Statistical analyses for health risk assessment

We used a linear mixed effects model to examine the short-term association between repeated measurements of PEF and daily PM_2.5_ total mass and five chemical species. We fitted the model assuming a linear exposure-response association and incorporated each of the chemical species at a time in the model with adjustment of the PM_2.5_ mass [19]. We also adjusted for sex, baseline age in 2014, body mass index (BMI, weight in kilograms divided by the square of height in meters) as both linear and quadratic terms, secondhand smoking status, and air cleaner use at home, fever as a binary variable of 1 if the daily body temperature was above 38.0 °C, the average daily mean temperature on the current day and the preceding day, daily mean relative humidity (the current day), a time trend within a year (day-of-season), and year as an indicator variable. We applied natural cubic B-splines for mean temperature and day-of-season with degrees of freedom (DF) of 3 and 5, respectively, and used a linear term for relative humidity, based on the results of the likelihood ratio test (Supplementary Materials, Table S2). In addition, we incorporated random intercepts for each subject and the 1st order residual autocorrelation to take into account the between-subject variations and residual serial correlations. The choice of the serial correlation structure was guided by the Akaike information criteria and Bayesian information criteria (Supplementary Materials, Table S3).

Furthermore, we used a generalized linear mixed effects model to examine the associations for asthma worsening episodes, incorporating the same potential confounders described above in the model. All analyses were performed separately for the morning and evening PEFs, and for each city.

The delayed effects of this association were also investigated. Since the 24-h PM_2.5_ samples were collected at noon between the morning and evening PEF measurements, we applied different lag days to the morning and evening PEFs to avoid using any post-exposure to the morning PEFs. Therefore, the delayed effects for the morning PEF were estimated on the preceding day (lag1) and the average cumulative exposures during the two preceding days (clag1–2) and three preceding days (clag1–3), respectively. Those for the evening PEF were estimated on the current day (lag0) and the average cumulative exposure on the current day and the preceding day (clag0–1), and up to two preceding days (clag0–2), respectively. No cumulative exposure was calculated if samples were unavailable for the lag periods.

We identified a few extremely high concentrations that could largely influence the fit of the statistical models. These observations were excluded from analysis. The influential points specifically included OC (> 20 μg/m^3^) and EC (> 3 μg/m^3^) on March 22, 2015, and NH_4_^+^ (> 7 μg/m^3^) on May 6, 2016, in Nagasaki city, and EC (> 8 μg/m^3^) on May 16, 2015, in Isahaya city (Supplementary Materials, Figures S2–S3).

### Sensitivity analysis

To confirm the robustness of the results, we performed sensitivity analysis. First, we repeated fitting the above-mentioned models with no adjustment of PM_2.5_ total mass and estimated the associations for each chemical species (i.e., a single pollutant model). Second, we added two variables for daily medication use (an inhaler and/or medication for internal use) for asthma to the single pollutant models, although roughly one-third of children for each city reported at least one time of their medication use. Lastly, we used a leave-one-out approach in which the subjects were excluded from the full dataset one at a time. We applied this approach to the linear mixed effects models to estimate the associations between daily morning PEF variations and two selected exposures at lag1 (i.e., sulfate for Nagasaki city and OC for Isahaya city).

We used the statistical software R version 3.6.3 (R Core Team, Vienna, Austria) for all analyses with several R packages, *nlme, lme4, splines, and lmtest*.

### Back-trajectory and cluster analysis

To investigate the pattern of the long-range transboundary PM_2.5_ in our study locations and the surrounding areas, we attempted to trace the air movement for each city during the PM_2.5_ sampling days described above. Specifically, we calculated a 72-h backward trajectory of air parcels for each day, representing a path of the air particles over specific grid points and the timing of reaching the sampling site. The trajectories were calculated based on the National Oceanic and Atmospheric Administration (NOAA) Hybrid Single Particle Lagrangian Integrated Trajectory model [20] (version 4.9; https://ready.arl.noaa.gov/HYSPLIT.php), running every hour from 03:00 to 02:00 UTC (12:00 to 11:00 local time) on each sampling day. In this computation, we incorporated the meteorological data from the Global Data Assimilation System (horizontal resolution of 0.5° × 0.5°) of the National Center for Environmental Prediction and assumed that the height of the endpoint was one-half of the mixing height over the planetary boundary layer. Next, we performed a cluster analysis and grouped the hourly trajectories into four clusters representing different paths and origins. The hourly clusters were converted to daily data, in which we calculated the proportions of the clusters in each day and chose the largest proportion as a dominant daily cluster. There were a few missing values for the clusters (6 and 8 days for Nagasaki city and Isahaya city, respectively) due to unavailable weather data from NOAA or the unclassified fifth cluster.

## Results

Table 1 shows the basic characteristics of the children with asthma observed in the baseline survey and summary statistics of the PEFs. More boys participated in this study (62.5% in Nagasaki city and 60.4% in Isahaya city), and the children from Isahaya city tended to be older with a higher BMI than those from Nagasaki city. In addition, a large number of children with asthma were exposed to secondhand smoke (41.7% in Nagasaki city and 30.6% in Isahaya city), while almost half never used air cleaners at home (50.0% and 51.0%, respectively). During the study period, the PEF were measured on average 170.0–194.8 times (days) per child. The average PEFs for individuals ranged from 215.3 L/min to 253.0 L/min and tended to be higher when measured in the evening than those measured in the morning.

**Table 1:** Summary statistics of the baseline survey and the self-measured peak expiratory flow (PEF) in children with asthma during March–June 2014–2016

Category	Nagasaki (*n* = 24)	Isahaya (*n* = 49)
Sex –boys^a^	15 (62.5)	29 (60.4)
Age in March 2014 (years)	7.1 (1.7)	8.7 (2.8)
Body weight (kg)	26.5 (7.8)	32.5 (12.3)
Body height (cm)	126.0 (11.0)	134.0 (16.2)
Body mass index (kg/m^2^)	16.4 (2.3)	17.4 (2.7)
Secondhand smoke^a^		
Yes	10 (41.7)	15 (30.6)
No	14 (58.3)	34 (69.4)
Use of air cleaner at home^a^		
Everyday	9 (37.5)	17 (34.7)
Sometimes	3 (12.5)	7 (14.3)
Never	12 (50.0)	25 (51.0)
Days with fever per child	1.3 (2.0)	1.9 (2.9)
Days with use of an inhaler per child	8.2 (26.9)	9.6 (34.5)
Days with medication for internal use per child	6.0 (19.3)	6.8 (16.8)
PEF (L/min)		
Morning PEF	215.3 (55.2)	247.6 (76.0)
Evening PEF	222.1 (53.8)	253.0 (76.2)
The number of PEF measurements in total (days)		
Morning PEF	4075	9468
Evening PEF	4365	9549
The number of PEF measurements per child (days)		
Morning PEF	170.0 (92.6)	193.2 (77.4)
Evening PEF	181.9 (87.9)	194.8 (73.6)
The number of PEF measurements per child (days)^b^		
Morning PEF	176.5 (1–282)	190.0 (0–276)
Evening PEF	170.0 (5–282)	209.0 (45–276)
Asthma worsening episodes based on PEF reduction > 15% (times)		
Morning PEF	4.2 (3.1)	4.7 (3.8)
Evening PEF	4.7 (3.0)	5.1 (3.7)
Asthma worsening episodes based on PEF reduction > 20% (times)		
Morning PEF	3.2 (2.9)	3.9 (3.7)
Evening PEF	3.6 (3.1)	3.9 (3.7)

Data are mean (SD), or n (%) with the superscript ‘a’; Data with the superscript ‘b’ show median and range (minimum to maximum)

Table 2 displays summary statistics of PM_2.5_ total mass, five chemical species of PM_2.5_, and weather variables in two cities. In general, the distributions of the PM_2.5_ mass, OC, and nitrate were relatively similar between the cities, while the concentrations of EC, sulfate, and ammonium were higher in Isahaya city than those in Nagasaki city. The correlation coefficients among the PM_2.5_ exposures were slightly different between the two cities, although a strong positive correlation between sulfate and ammonium was consistently observed in both cities (Table 3). Specifically, in Isahaya city, the PM_2.5_ mass and chemical species were highly correlated except for nitrate, and a high correlation was also observed between OC and EC, while no strong correlations among the species were observed in Nagasaki city. In addition, moderately high correlations were observed between EC and three inorganic ions (i.e., nitrate, sulfate, and ammonium) in Isahaya city, whereas they were relatively weak in Nagasaki city. Of note, the statistics are not directly comparable between the cities because of different sampling days and possible monthly variations in the exposures accordingly (Supplementary Materials, Table S1).

**Table 2:** Summary statistics of PM_2.5_ total mass and chemical species and weather factors during March–June 2014–2016

City/Variables	#days	Median (IQR)	Min	Max	Percent PM_2.5_ mass
Nagasaki (Mar–June)					
PM_2.5_ (μg/m^3^)	205	16.2 (11.5)	0.9	73.3	–
OC (μg/m^3^)	205	2.9 (2.0)	0.7	20.6	18.1
EC (μg/m^3^)	205	0.4 (0.2)	< 0.01	3.5	2.5
Nitrate (μg/m^3^)	205	0.8 (1.0)	0.1	9.4	6.8
Sulfate (μg/m^3^)	205	1.1 (1.9)	0.0	8.0	9.9
Ammonium (μg/m^3^)	205	0.4 (0.8)	0.0	7.8	4.4
Mean temperature (°C)	366	17.5 (4.7)^a^	3.2	26.2	–
Relative humidity (%)	366	73.3 (13.3)^a^	37.0	99.0	–
Isahaya (Mar–May)					
PM_2.5_ (μg/m^3^)	183	17.7 (9.8)	3.1	58.0	–
OC (μg/m^3^)	183	2.6 (1.8)	0.7	9.4	15.4
EC (μg/m^3^)	183	1.0 (0.8)	0.3	8.9	6.4
Nitrate (μg/m^3^)	183	0.5 (1.1)	< 0.01	10.0	5.2
Sulfate (μg/m^3^)	183	5.1 (3.6)	1.2	17.0	29.9
Ammonium (μg/m^3^)	183	2.2 (1.7)	0.4	8.1	13.0
Mean temperature (°C)	276	15.9 (4.3)^a^	3.2	23.3	–
Relative humidity (%)	276	69.7 (12.3)^a^	37.0	96.0	–

^a^ Mean (SD); *IQR* interquartile range

**Table 3:** Spearman correlation between daily concentrations of PM_2.5_ total mass and chemical species

	PM_2.5_	OC	EC	Nitrate	Sulfate	Ammonium
Nagasaki						
PM_2.5_ mass	1	0.50	0.54	0.21	0.57	0.67
OC		1	0.49	0.15	0.46	0.49
EC			1	0.08	0.26	0.38
Nitrate				1	0.42	0.29
Sulfate					1	0.80
Ammonium						1
Isahaya						
PM_2.5_ mass	1	0.77	0.77	0.44	0.88	0.88
OC		1	0.80	0.41	0.56	0.59
EC			1	0.62	0.63	0.72
Nitrate				1	0.31	0.50
Sulfate					1	0.96
Ammonium						1

*EC* elemental carbon; *OC* organic carbon

**Fig. 1:**
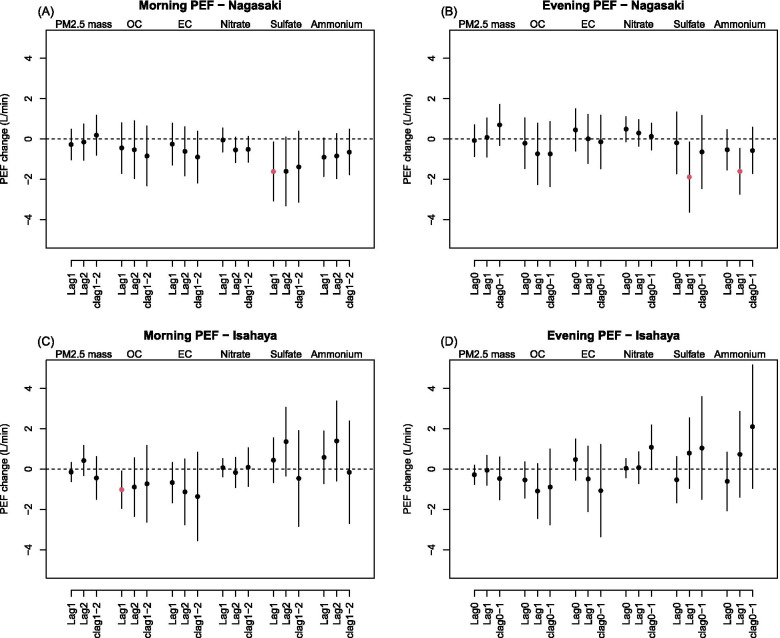
Changes in the daily peak expiratory flow (PEF) per interquartile range (IQR) increase in concentrations of PM_2.5_ total mass and five chemical species in Nagasaki city (**A** and **B**) and Isahaya city (**C** and **D**), estimated by the linear mixed effects model adjusting for potential confounders and PM_2.5_ mass. Different lag days were applied to the morning PEFs (**A** and **C**) on the preceding day (lag1), the cumulative exposure during two preceding days (clag1–2), and the cumulative exposure during three preceding days (clag1–3) and the evening PEF (**B** and **D**) on the current day (lag0), the cumulative exposure on the current day and the preceding day (clag0–1), and the cumulative exposure up to two preceding days (clag0–2). The vertical bars indicate 95% confidence intervals. OC: organic carbon; EC: elemental carbon

Figure 1 shows daily morning and evening PEF changes per interquartile range (IQR) increase in PM_2.5_ exposures by lag days. We found that higher average concentrations of sulfate and ammonium at clag0–1 (from the preceding day to the current day) were associated with lower levels of evening PEF in Nagasaki city after adjusting for PM_2.5_ mass and other confounders (Fig. 1-B). The estimated changes in the evening PEF per IQR increase were − 1.88 L/min (95% confidence interval (CI): − 3.62, − 0.14) and − 1.60 L/min (95% CI: − 2.73, − 0.47) respectively. A negative association for sulfate was also observed in the morning PEF at lag1 (the preceding day) in the same city (Fig. 1-A). In Isahaya city, we found that higher OC at lag1 was negatively associated with morning PEF after adjusting for PM_2.5_ mass (Fig. 1-C). The estimated change in the morning PEF was − 1.02 L/min (95% CI: − 1.94, − 0.09) per IQR increase. These results were generally consistent in single pollutant models with no adjustment of the PM_2.5_ mass (Supplementary Materials, Figure S4). No evidence was found for the total mass of PM_2.5_ and other chemical species.

**Fig. 2:**
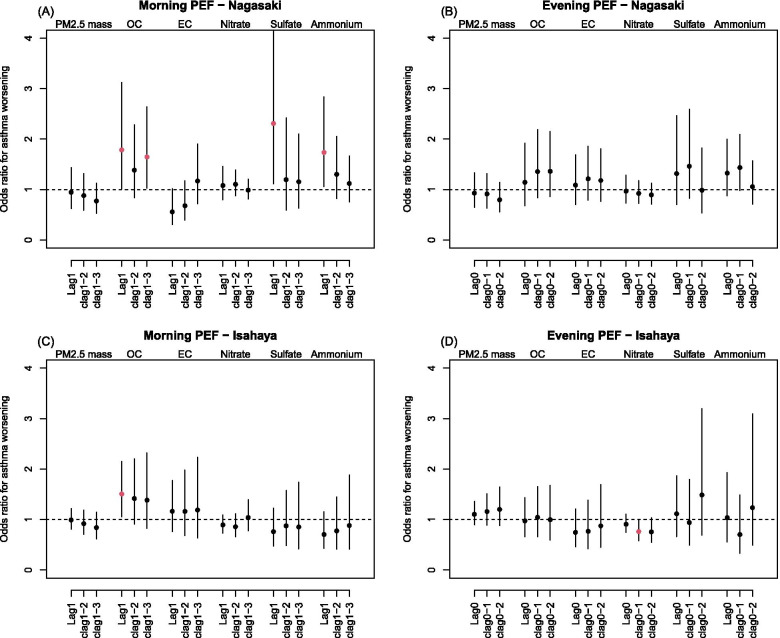
Odds ratios for the asthma worsening episodes per interquartile range (IQR) increase in concentrations of PM_2.5_ total mass and five chemical species in Nagasaki city (**A** and **B**) and Isahaya city (**C** and **D**), estimated by the generalized linear mixed effects model adjusting for potential confounders and PM_2.5_ mass. The episode was defined if the percent reduction in daily peak expiratory flow (PEF) from the personal best monthly PEF was > 15% within a rolling 1-week window. Different lag days were applied to the morning PEFs (**A** and **C**) on the preceding day (lag1), the cumulative exposure during two preceding days (clag1–2), and the cumulative exposure during three preceding days (clag1–3) and the evening PEF (**B** and **D**) on the current day (lag0), the cumulative exposure on the current day and the preceding day (clag0–1), and the cumulative exposure up to two preceding days (clag0–2). The vertical bars indicate the 95% confidence intervals. OC: organic carbon; EC: elemental carbon

Figure 2 displays the odds ratios (OR) for the asthma worsening episodes, defined by the PEF reduction > 15% from personal monthly maximum, per IQR increase in PM_2.5_ exposures by lag days. We observed consistent findings with those for daily PEF variations above, in which there were higher odds of asthma worsening in the morning for sulfate (OR = 2.31; 95% CI: 1.12, 4.77) and ammonium (OR = 1.73; 95% CI: 1.06, 2.84) in Nagasaki city and OC (OR = 1.51; 95% CI: 1.06, 2.15) in Isahaya city, at lag1 for all three chemical species after adjusting for PM_2.5_ mass and other confounders (Fig. 2-A and 2-C). However, these results were insignificant in single pollutant models with no adjustment of the PM_2.5_ mass (Supplementary Materials, Figure S5), and inconsistent results were estimated based on another cut-off of 20% PEF reduction (Supplementary Materials, Figures S6-S7).

These results for the health risk assessment were fairly robust (Supplementary Materials, Figures S8-S9) and not highly influenced by a particular child from the leave-one-out approach in the sensitivity analysis (Supplementary Materials, Figure S10).

**Fig. 3:**
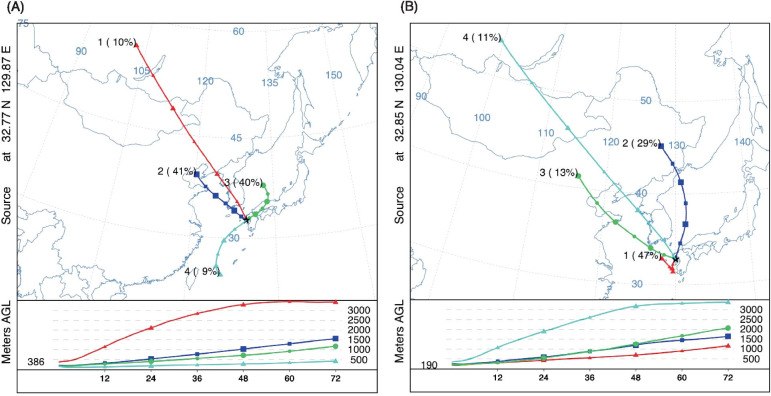
Cluster paths of the long-range transboundary PM_2.5_ calculated by the 72-h backward trajectories over sampling days in the study locations (**A**: Nagasaki city, **B**: Isahaya city)

Figure 3 depicts the results of the back-trajectory and cluster analyses over sampling days of each city based on 4790 and 4392 hourly trajectories in Nagasaki city and Isahaya city, respectively. We found that the contributions of a long-range transport from Russia that passed through Mongolia and North China were estimated in similar proportions between the cities (cluster #1 of 10% [17.2 ± 9.24 μg/m^3^] for Nagasaki city and cluster #4 of 11% for Isahaya city [22.1 ± 11.4 μg/m^3^]). However, the patterns of the clusters and the contributions of other clusters were inconsistent across cities. In Nagasaki city, the trajectories originating from Northeast China, where the largest industrial production and manufacturing have been developed, were fairly dominant (cluster #2, 41%), followed by those from local sources (cluster #3, 40%). In contrast, in Isahaya city, the trajectories originating from Northeast China contributed fairly small (cluster #3, 13%), but those from local sources were highly dominant (cluster #1, 47%).

**Fig. 4:**
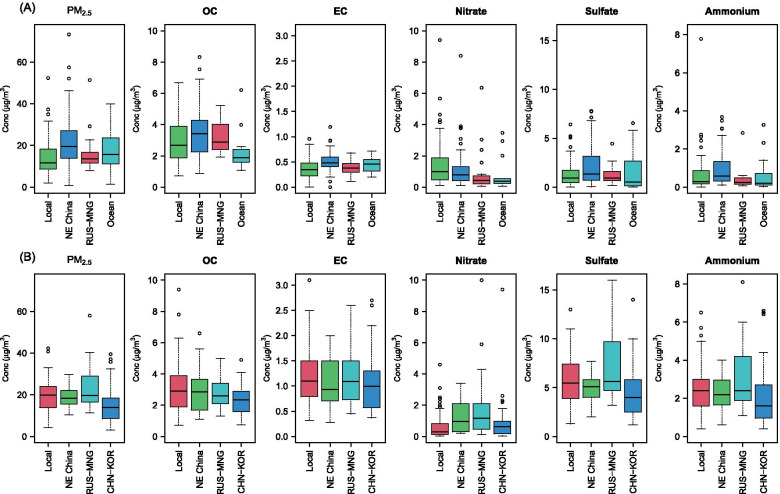
Box plots for PM_2.5_ total mass and five chemical species by clusters in (**A**) Nagasaki city and (**B**) Isahaya city. NE China: Northeast China. RUS-MNG: Russia passed through Mongolia and North China. CHN-KOR: North China passed through East of the Korea peninsula. Conc: concentration

Figure 4 presents concentrations of the PM_2.5_ mass and chemical species by daily clusters in each city. In Nagasaki city, the concentrations were consistently highest for the cluster of Northeast China origins (e.g., 21.6 ± 11.7 μg/m^3^ for PM_2.5_ mass) across all the PM_2.5_ exposures, except for nitrate. In contrast, higher concentrations were observed in days by the local cluster in Isahaya city (e.g., 19.4 ± 7.53 μg/m^3^ for PM_2.5_ mass), except for nitrate, although the concentrations for the local cluster were analogous to those for other two pathways from Northeast China and Russia that passed through Mongolia.

## Discussion

The present study indicates that a decline in lung function was associated with increased exposure to sulfate, ammonium, and OC fractions of PM_2.5_, but not with PM_2.5_ mass, in the multiyear panels of children with asthma who resided in two urban areas in Nagasaki prefecture, Japan. We observed that concentrations of the significant chemical species were higher in days largely contributed by the path of Northeast China origin (for sulfate and ammonium) or both the same path and local sources (for OC) than by other clusters, as suggested by the back-trajectory and cluster analyses.

Existing evidence about the differential effects of PM_2.5_ fractions on children’s lung function has been inconclusive, although it appears more evidence suggests that EC and black carbon are associated with asthma exacerbations among children [21]. We found no evidence for EC and nitrate in this study; however, our findings were consistent with those of some previous studies. For example, exposure to sulfate, ammonium, EC, and/or OC fractions of PM_2.5_ was adversely associated with pediatric emergency department visits for asthma and wheezing [22], and upper respiratory infections [23] among children in the United States. A panel study in Southern China also reported an association between OC and lung function decline in healthy school children [24]. Our previous study in the same city (Isahaya only) for adults with asthma showed consistent findings that severe respiratory decline was associated with increased sulfate and OC fractions of PM_2.5_ [7].

Different environmental conditions, characterized by geographical terrain, seasonal features (e.g., monsoon and seasonal prevailing wind), or major sources of air pollution that could potentially influence the fractions of PM_2.5_, might magnify the effects of certain chemical species on health for each study area. The secondary pollutants (sulfate and ammonium) and the mixed basis (OC) in our study were found to be associated with lung function decrements among children with asthma. We speculate that the effects of transboundary air pollution in our study area played a role in the negative associations in the particular season (spring) because relatively large contributions to the path from Northeast China to Nagasaki city were estimated for those chemical species by back-trajectory and cluster analyses. A previous study conducted in Fukuoka and Fukue Island, both located approximately 100 km away from the city of Nagasaki, also reported consistent observations from 2010 to 2013 that sulfate and OC were the largest contributing components to PM_2.5_, and greatly dominated by the inflow of long-range transported aerosols [25]. A previous study on children with asthma in Nagasaki prefecture (the same but larger panel as the present study without PM_2.5_ composition sampling) also reported PEF reduction with transboundary air pollution, represented by AD events detected by light detection and ranging and suspended particulate matter [26]. Consistent evidence for the AD events associated with pediatric emergency department visits due to bronchial asthma was also reported for school children during the spring in Nagasaki prefecture [27].

Given the anticipation of the potential effects of long-range transboundary air pollution in our study areas, we initially hypothesized that the results from the two neighboring cities of this study would be identical enough to be combined. However, the estimated trajectories representing prevailing paths and clusters for the PM_2.5_ mass and chemical species were different between the cities (i.e., the largest contribution from the path of Northeast China origin in Nagasaki city, whereas almost even contributions from the two paths of Northeast China and local origins in Isahaya city). We therefore performed all analyses separately for each city and observed that the chemical species of PM_2.5_, which were associated with declines in lung function, also differed by city (i.e., sulfate and ammonium in Nagasaki city and OC in Isahaya city).

Several possible explanations may support this discrepancy. First, the specific days of the PM_2.5_ sampling between the two cities were not identical, although the PM_2.5_ samples were collected in the same season and year. In particular, the proportion of AD events that occurred during the sampling days was higher in Nagasaki city than in Isahaya city (Supplementary Materials, Table S1), which could result in different patterns of estimated trajectories. In addition, the different elements of topography between the two cities might contribute to the discrepancy. Nagasaki city is adjacent and widely open to the East China Sea, where the westerly wind could constantly flow into the city with no major obstacles (Figure S1). In contrast, Isahaya city is located slightly more inland sheltered by a range of mountains that may weaken the influence of the westerly wind and long-range transport air pollution. The city also has a more robust industrial sector promoted since the 2010s. Our previous study in the same city suggested an association between severe respiratory declines among adults with asthma and secondary sulfate emission source, which was likely driven by OC [7]. Other possibilities exist regarding the different health risks estimated between cities. Although we attempted to adjust for potential individual and time-varying confounders in the models, some other factors related to different susceptibility in children between cities might play a role in modifying the health risks that we could not fully consider in this study. Lastly, the difference in specific details in sampling and analysis methods for the PM_2.5_ exposure data between two sites might partly influence the discrepancy, although we believe that the methods and filters were comparable enough for the health risk assessment to observe the short-term associations.

The biological plausibility of the short-term effects of PM_2.5_ on asthma exacerbation has been well described, coupled with the potential pathways through respiratory tract injury, airway inflammation, oxidative stress, airway hyper-responsiveness, allergic sensitization, or activation of sensory nerves in the respiratory tract [1, 21]. We believe that the same mechanisms could apply to the adverse effects of sulfate, ammonium, and OC fractions of PM_2.5_, on lung function in children with asthma. Furthermore, our findings of the exposure-response associations for these fractions provide evidence that even low levels of the components of PM_2.5_ could trigger airway inflammation. In fact, the annual PM_2.5_ levels in our study areas have been relatively low and observed around the Japanese National Ambient Air Quality Standard (NAAQS) (annual mean of 15 μg/m^3^ and daily mean of 35 μg/m^3^), with a small number of days exceeding the daily standard (i.e., less than 5% for a year during our study periods) [28].

Nevertheless, our results should be interpreted with caution from a clinical perspective because it is arguable whether the PEF reduction of 1–2 L/min per IQR increase in the PM_2.5_ fractions could provide clinical implications for children with asthma. In addition, we observed that the odds of asthma worsening episodes associated with PM_2.5_ were fairly sensitive to cut-offs to define an episode. In particular, the estimated odds based on the cut-off of 20% PEF reduction were counterintuitive and inconsistent with those for another cut-off of 15% PEF reduction. We believe that the unexpected results for the cut-off of 20% may be due to well-guided self-management of the asthma action plan by the children with asthma and their parents enrolled in our study. Given that the PEF reduction > 20% for more than two days has been recommended by the Global Initiative for Asthma as a clinically important criterion for initiating the use of controller medication [17], our findings for the less strict cut-off of 15% PEF reduction suggest that the fractions of PM_2.5_ could be implicated in the subclinical status of asthma worsening among children.

A major strength of this study was that we obtained a large number of daily PEF measurements over three years in the spring, while most previous panel studies investigating the associations with PM_2.5_ exposures have been designed to collect lung function measurements over multiple days or a few months [24, 29–32]. The large number of PEF measurements in our study could increase the statistical power to detect a smaller effect size for the exposure-response associations among children with asthma, who are one of the most vulnerable populations to air pollution exposure. In addition, given varying levels of PM_2.5_, the inclusion of the three years of data in our study could lead to a more generalizable conclusion for the associations in the study areas.

This study had several limitations. First, we collected daily self-measured PEFs, which were not measured under the supervision of medical staff. Although we trained the children with asthma and their parents and excluded the training periods (used to become accustomed to the measuring device) from our analyses, the measurement error might be greater than that under supervision. In addition, the self-measurement performance could naturally be improved over time through daily repetition. We included the day of the year in the models to adjust for the possible time trends of the self-measured PEFs. Second, we collected ambient PM_2.5_ exposures derived from fixed-site monitors, but not personal exposures. Some previous studies have reported weaker associations between children’s lung function and ambient air pollution exposure than those for personal exposures, presumed to be a superior method of exposure assessment [29, 33]. However, this possible misclassification in our study would be non-differential, leading to bias towards the null [34]. Third, we fitted the models including a single chemical species with no adjustment for other species, although the associations for each chemical species were estimated after adjusting for PM_2.5_ total mass. Lastly, our study did not take into account seasonal aeroallergens such as pollen because of data unavailability, although pollen could potentially be associated with asthma worsening, independently or synergistically with fine particulate matter [35].

## Conclusions

This study provides evidence of the differential effects of PM_2.5_ fractions on lung function among children with asthma in urban areas where the Japanese NAAQS has been nearly met. We suggest that continuous efforts to promote mitigation actions and awareness of the hazards of transboundary air pollution (e.g., reducing outdoor activities on days when higher levels of PM_2.5_ are forecast) are warranted to reduce the health burden, particularly among susceptible children with asthma. Furthermore, international collaborative research in East Asia is needed to address the issue of transboundary particulate matter air pollution based on systematic investigations and consequently protect vulnerable populations from exposure to air pollution.

## Supplementary Information

**Additional file 1.**

## Data Availability

The data that support the findings of this study are available from the Ministry of the Environment in Japan, but restrictions apply to the availability of these data, which were used under license for the current study, and so are not publicly available. Data are however available from the authors upon reasonable request and with permission of the Ministry of the Environment, Japan.

## Abbreviations

PM_2.5_: Fine particulate matter
PEF: Peak expiratory flow
OC: Organic carbon
EC: Elemental carbon
IQR: Interquartile range
OR: Odds ratio
AD: Asian Dust
JMA: Japanese Meteorological Agency
DL: Detection limit
BMI: Body mass index
NOAA: National Oceanic and Atmospheric Administration
NAAQS: National Ambient Air Quality Standard
