# Supplementary material for: Respiratory function declines in children with asthma associated with chemical species of fine particulate matter (PM2.5) in Nagasaki, Japan
Source: Environ Health. 2021 Oct 21;20:110. doi: 10.1186/s12940-021-00796-x (PMC8529805; doi:10.1186/s12940-021-00796-x)
Supplement: Supplementary file 1 — Additional file 1. [file 12940_2021_796_MOESM1_ESM.docx]

**Supplementary Materials**

**Table of contents**

PM_2.5_ sampling and analysis methods for each city

Table S1. The number of sampling days for PM_2.5_ total mass and its chemical species

Table S2. Model selection by the likelihood ratio test (LRT) among models fitted with different degrees of freedom (DF) of natural cubic B-splines for three variables

Table S3. Model selection guided by Akaike Information Criteria (AIC) and Bayesian Information Criteria (BIC) among models fitted with different serial correlation structures for the within-subject errors

Figure S1. (A) Study area in the red rectangle and (B) the enlarged area including locations of Nagasaki University Hospital and Isahaya General Hospital.

Figure S2. Time-series plots of the daily mean concentrations of PM2.5 total mass and its chemical species in Nagasaki city

Figure S3. Time-series plots of the daily mean concentrations of PM2.5 total mass and its chemical species in Isahaya city

Figure S4. Changes in the daily peak expiratory flow (PEF) per an interquartile range (IQR) increase in the PM_2.5_ exposures, with no adjustment of PM_2.5_ mass.

Figure S5. Odds ratios for the asthma worsening episodes, defined by the peak expiratory flow (PEF) reduction >15% from personal monthly maximum, per an interquartile range (IQR) increase in the PM_2.5_ exposures, with no adjustment of PM_2.5_ mass.

Figure S6. Odds ratios for the asthma worsening episodes, defined by the peak expiratory flow (PEF) reduction >20% from personal monthly maximum, per an interquartile range (IQR) increase in the PM_2.5_ exposures, adjusting for potential confounders and PM_2.5_ mass.

Figure S7. Odds ratios for the asthma worsening episodes, defined by the peak expiratory flow (PEF) reduction >20% from personal monthly maximum, per an interquartile range (IQR) increase in the PM_2.5_ exposures, with no adjustment of PM_2.5_ mass.

Figure S8. Changes in the daily peak expiratory flow (PEF) per an interquartile range (IQR) increase in the PM_2.5_ exposures, with additional adjustment of medication use for asthma.

Figure S9. Odds ratios for the asthma worsening episodes, defined by the peak expiratory flow (PEF) reduction >15% from personal monthly maximum, per an interquartile range (IQR) increase in the PM_2.5_ exposures, with additional adjustment of medication use for asthma.

Figure S10. Results of the leave-one-out approach by children’s ID.

**PM_2.5_ sampling and analysis methods for each city**

In Nagasaki city, we used a low-volume sampler with a flow rate of 16.7 L/min for mass and carbonaceous species and 10.0 L/min for ionic species and used U.S. Environmental Protection Agency (EPA) Compendium Method IO-4.2 for PM2.5 component analyses. We collected the PM2.5 mass by weighing polytetrafluoroethylene (PTFE) filters (1-μm pore size, Pall Life Sciences) with a microbalance (AND HM-202, precision 10^-2^ mg). The carbonaceous species were collected using Quartz filters (Pallflex Membrane Filters, Pall Life Sciences) and analyzed using thermal-optical transmittance with the National Institute of Occupational Safety and Health 5040 method (Thermal-Optical Carbon Aerosol Analyzer, Sunset Laboratory). Also, the ionic species were collected using extra Zeﬂuor membrane ﬁlters (1-μm pore size, Pall Life Sciences) and analyzed using ion chromatography (Thermo Scientific Dionex ICS-1100). All filters had a diameter of 47 mm.

In Isahaya city, we used low-volume samplers (SIBATA Sciences LV-250 for 2014 and Thermo Fisher Scientific 2025 Sequential Air Sampler for 2015–2016), based on U.S. EPA Federal Equivalent Method (FEM), with the flow rate of 16.7 L/min. We collected the PM2.5 mass by weighing PTFE tape filters (KFT-730, KIMOTO). The carbonaceous species were collected using Quartz filters (Pallflex Air Monitoring 2500 QAT-UP filters) and analyzed using the thermal-optical reflectance method (Carbon Aerosol Laboratory Instrument Model4, Sunset Laboratory). Also, the ionic species were collected using extra Quartz filters (Pallflex Air Monitoring 2500 QAT-UP filters) and analyzed using ion chromatography (Thermo Scientific Dionex ICS-1600). All filters had a diameter of 47 mm.

Table S1. The number of sampling days for PM_2.5_ total mass and its chemical species and Asian Dust event observations^a^ during the sampling days

| Sampling site | Month | 2014 | 2015 | 2016 |
| --- | --- | --- | --- | --- |
| Nagasaki city |  |  |  |  |
|  | March | 9 (0) | 24 (1) | 22 (0) |
|  | April | 16 (0) | 19 (0) | 23 (2) |
|  | May | 16 (5) | 15 (0) | 20 (0) |
|  | June | 16 (0) | 13 (2) | 12 (0) |
| Isahaya city |  |  |  |  |
|  | March | 31 (0) | 30 (1) | 31 (0) |
|  | April | NA | 30 (0) | 30 (2) |
|  | May | NA | 31 (1) | NA |
|  | June | NA | NA | NA |

^a^The information about Asian Dust events in parentheses were observed by Japanese Meteorological Agency (<https://www.data.jma.go.jp/gmd/env/kosahp/kosa_data_index.html>) at the Fukuoka site, the nearest one to Nagasaki prefecture among multiple observatories across the country.

Table S2. Model selection by the likelihood ratio test (LRT) among models fitted with different degrees of freedom (DF) of natural cubic B-splines for three variables (i.e., mean temperature, relative humidity, and day-of-season)

| City | Variable | DF | Model comparison | P-value for LRT^a^ |
| --- | --- | --- | --- | --- |
| Nagasaki | Mean temperature | Linear | Linear vs. df=2 | 0.010 |
|  |  | 2 | df=2 vs. df=3 | 0.018 |
|  |  | 3 | df=3 vs. df=4 | 0.026 |
|  |  | 4 | df=4 vs. df=5 | 0.016 |
|  |  | 5 |  |  |
|  | Relative humidity | Linear | Linear vs. df=2 | 0.061 |
|  |  | 2 | df=2 vs. df=3 | 0.064 |
|  |  | 3 | df=3 vs. df=4 | 0.062 |
|  |  | 4 | df=4 vs. df=5 | 0.040 |
|  |  | 5 |  |  |
|  | Day-of-season | Linear | Linear vs. df=2 | 0.007 |
|  |  | 2 | df=2 vs. df=3 | 0.013 |
|  |  | 3 | df=3 vs. df=4 | 0.012 |
|  |  | 4 | df=4 vs. df=5 | 0.008 |
|  |  | 5 | df=5 vs. df=6 | 0.002 |
|  |  | 6 | df=6 vs. df=7 | 0.003 |
|  |  | 7 | df=7 vs. df=8 | 0.007 |
|  |  | 8 |  |  |
| Isahaya | Mean temperature | Linear | Linear vs. df=2 | 0.043 |
|  |  | 2 | df=2 vs. df=3 | 0.047 |
|  |  | 3 | df=3 vs. df=4 | 0.071 |
|  |  | 4 | df=4 vs. df=5 | 0.062 |
|  |  | 5 |  |  |
|  | Relative humidity | Linear | Linear vs. df=2 | 0.085 |
|  |  | 2 | df=2 vs. df=3 | 0.168 |
|  |  | 3 | df=3 vs. df=4 | 0.049 |
|  |  | 4 | df=4 vs. df=5 | 0.100 |
|  |  | 5 |  |  |
|  | Day-of-season | Linear | Linear vs. df=2 | 0.002 |
|  |  | 2 | df=2 vs. df=3 | 0.037 |
|  |  | 3 | df=3 vs. df=4 | 0.014 |
|  |  | 4 | df=4 vs. df=5 | 0.006 |
|  |  | 5 | df=5 vs. df=6 | 0.045 |
|  |  | 6 | df=6 vs. df=7 | 0.017 |
|  |  | 7 | df=7 vs. df=8 | 0.038 |
|  |  | 8 |  |  |

^a^A linear mixed effects model was performed, including the morning PEF (L/min) and the selected exposure (i.e., sulfate at lag1 in Nagasaki city and OC at lag1 in Isahaya city), adjusting for potential confounders and PM_2.5_ total mass at lag1.

Table S3. Model selection guided by Akaike Information Criteria (AIC) and Bayesian Information Criteria (BIC) among models fitted with different serial correlation structures for the within-subject errors

| City | Models^a^ with a serial correlation structure | Degree of freedom | AIC | BIC |
| --- | --- | --- | --- | --- |
| Nagasaki |  |  |  |  |
|  | Independent | 34 | 20507.9 | 20703.2 |
|  | Compound symmetry | 35 | 20509.9 | 20710.9 |
|  | Autoregressive (AR1) | 35 | 19312.2 | 19513.2 |
|  | Moving average (MA2) | 36 | 19550.8 | 19757.5 |
|  | Gaussian spatial^b^ | 35 | 19760.0 | 19960.9 |
|  | Rational quadratic spatial^b^ | 35 | 19405.6 | 19606.6 |
| Isahaya |  |  |  |  |
|  | Independent | 34 | 56611.5 | 56840.7 |
|  | Compound symmetry | 35 | 56613.5 | 56849.5 |
|  | Autoregressive (AR1) | 35 | 53353.4 | 53589.4 |
|  | Moving average (MA2) | 36 | 54038.7 | 54281.4 |
|  | Gaussian spatial^b^ | 35 | 54554.9 | 54790.8 |
|  | Rational quadratic spatial^b^ | 35 | 53640.1 | 53876.1 |

^a^Models to estimate the association between morning PEF and the selected exposure (i.e., sulfate at lag1 in Nagasaki city and OC at lag1 in Isahaya city), adjusting for potential confounders and PM_2.5_ mass at lag1

^b^The ‘date’ variable was used to specify the distance for both the spatial correlations.


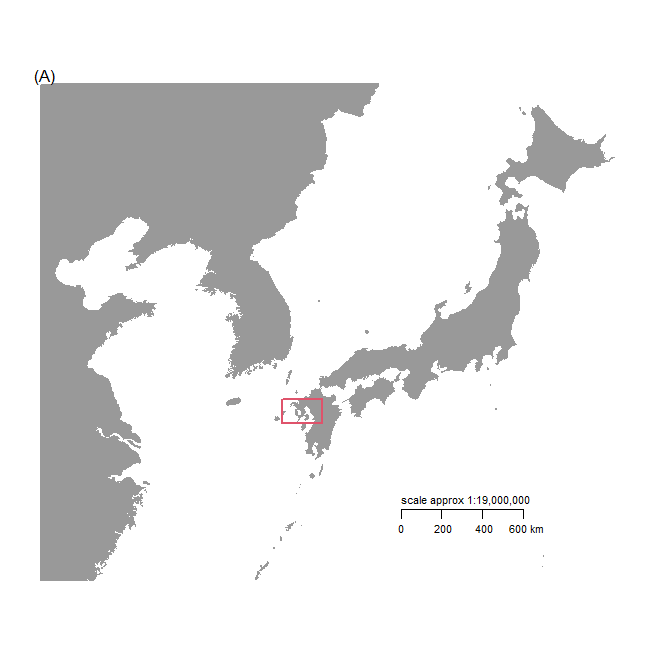


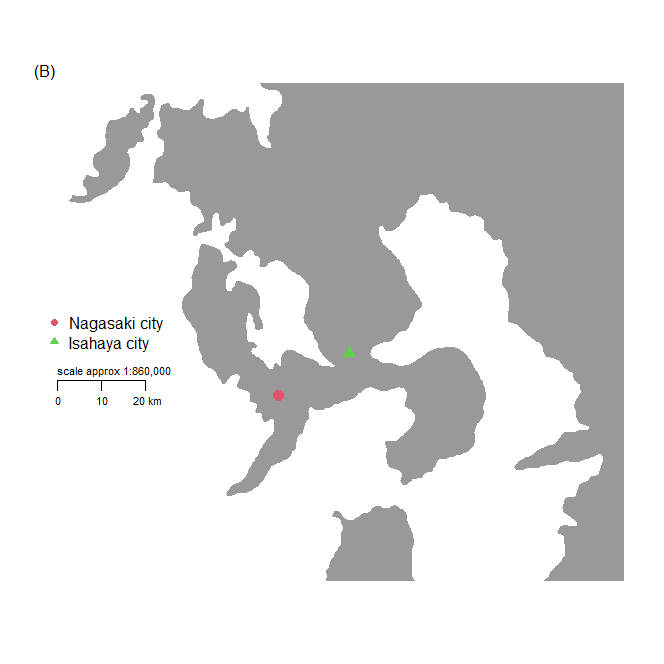


Figure S1. (A) Study area in the red rectangle and (B) the enlarged area including locations of Nagasaki University Hospital (red circle) and Isahaya General Hospital (green triangle).


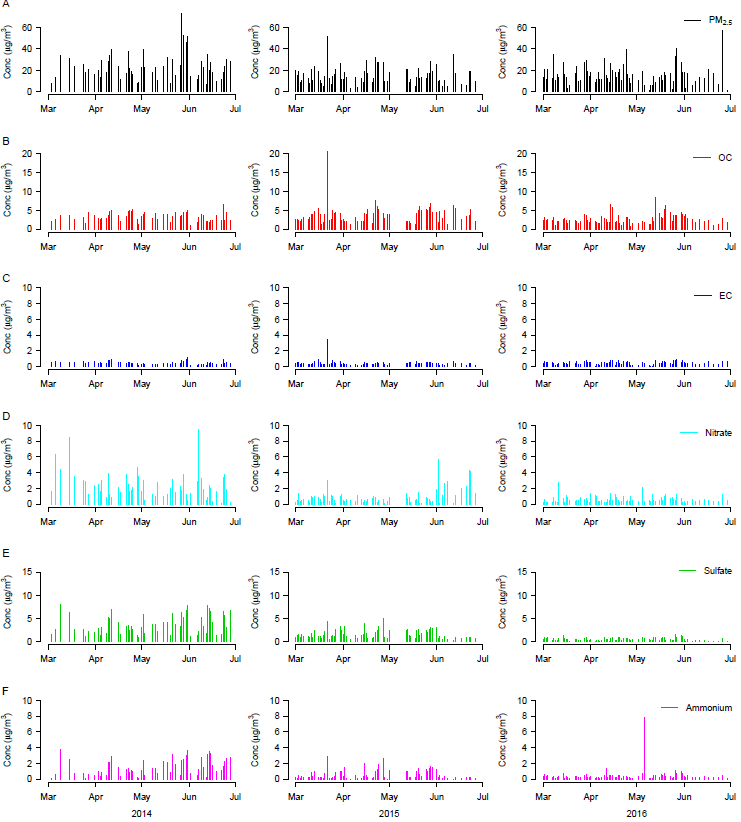


Figure S2. Time-series plots of the daily mean concentrations of PM_2.5_ total mass and its chemical species in Nagasaki city Mar–June 2014–2016. OC: organic carbon; EC: elemental carbon; Conc: concentration.


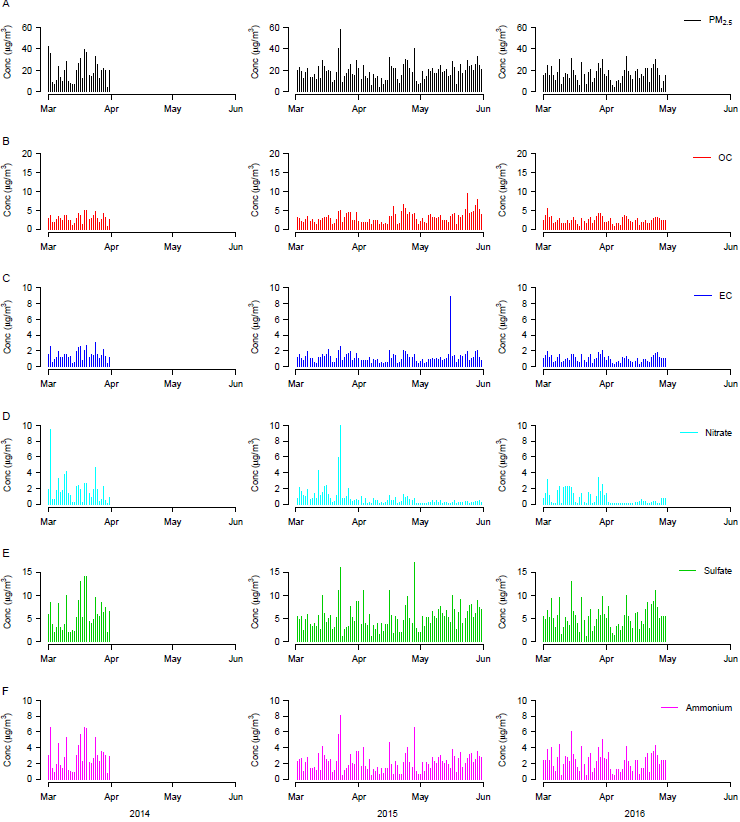


Figure S3. Time-series plots of the daily mean concentrations of PM_2.5_ total mass and its chemical species in Isahaya city Mar–June 2014–2016. OC: organic carbon; EC: elemental carbon; Conc: concentration.


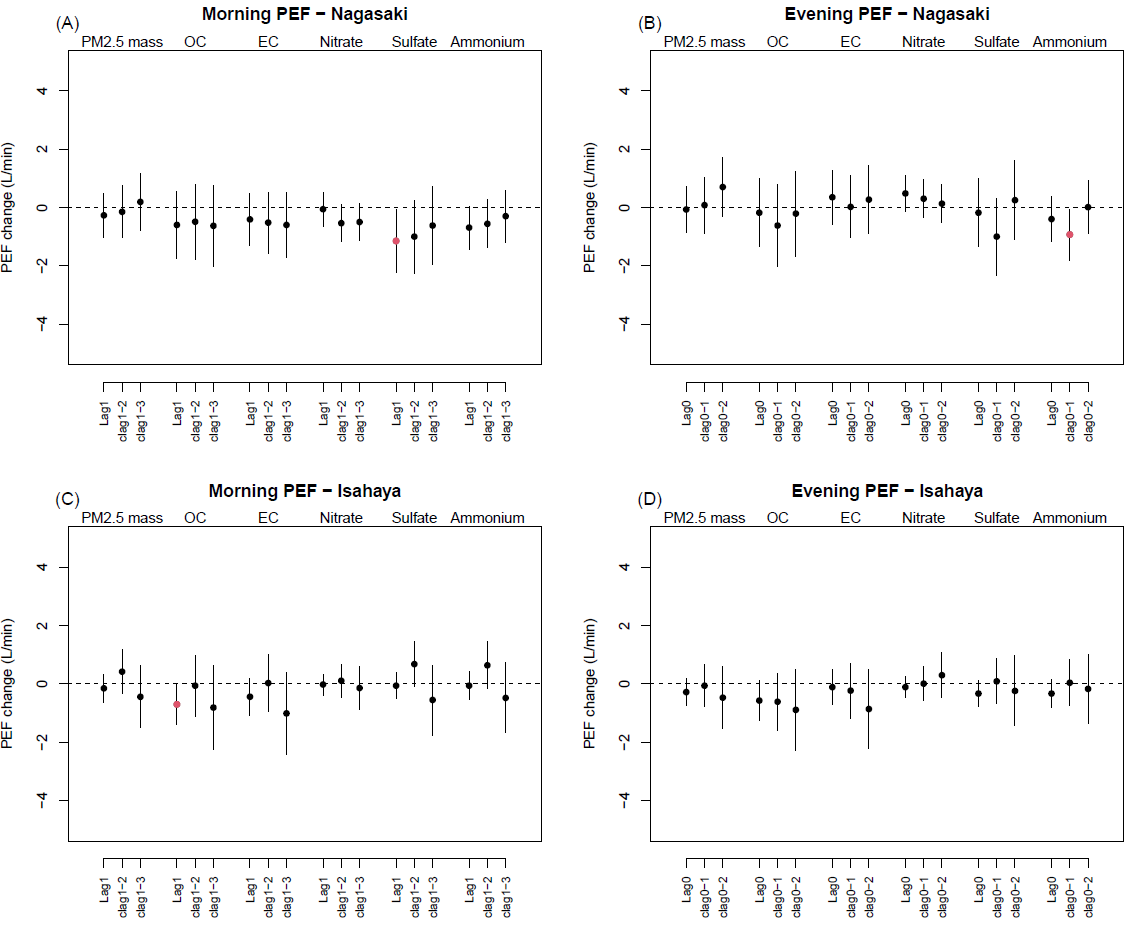


Figure S4. Changes in the daily peak expiratory flow (PEF) per an interquartile range (IQR) increase in concentrations of PM_2.5_ total mass and five chemical species in Nagasaki city (A and B) and Isahaya city (C and D), estimated by the linear mixed effects model with no adjustment of PM_2.5_ mass. Different lag days were applied to the morning PEFs (A and C) on the preceding day (lag1), the cumulative exposure during two preceding days (clag1–2), and the cumulative exposure during three preceding days (clag1–3) and the evening PEF (B and D) on the current day (lag0), the cumulative exposure on the current day and the preceding day (clag0–1), and the cumulative exposure up to two preceding days (clag0–2). OC: organic carbon; EC: elemental carbon.


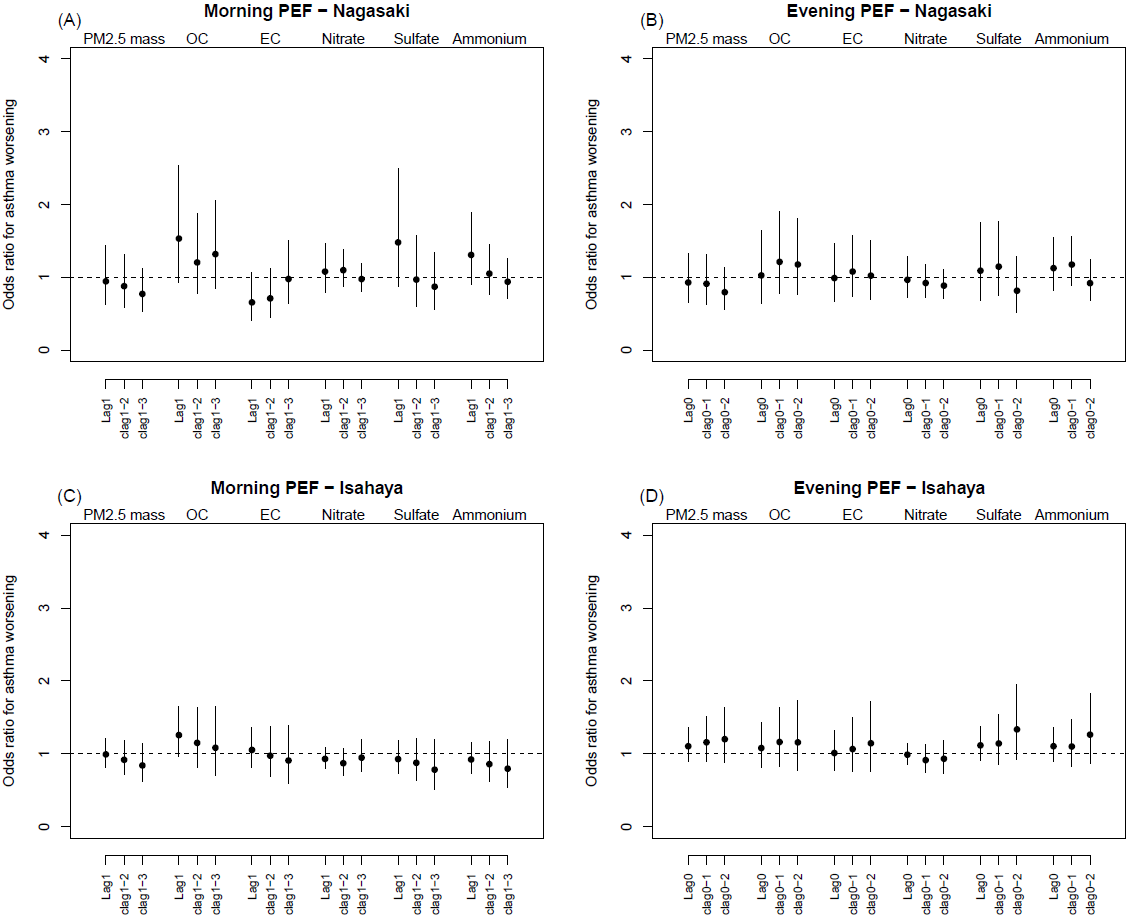


Figure S5. Odds ratios for the asthma worsening episodes, defined by the peak expiratory flow (PEF) reduction >15% from personal monthly maximum, per an interquartile range (IQR) increase in the PM_2.5_ exposures, estimated by the generalized linear mixed effect models with no adjustment of PM_2.5_ mass in Nagasaki city (A and B) and Isahaya city (C and D). Different lag days of the exposures were applied to the morning PEFs (A and C) on the preceding day (lag1), the two preceding days (clag1–2), and the three preceding days (clag1–3) and the evening PEF (B and D) on the current day (lag0), the current day and the preceding day (clag0–1), and up to two preceding days (clag0–2). OC: organic carbon; EC: elemental carbon.


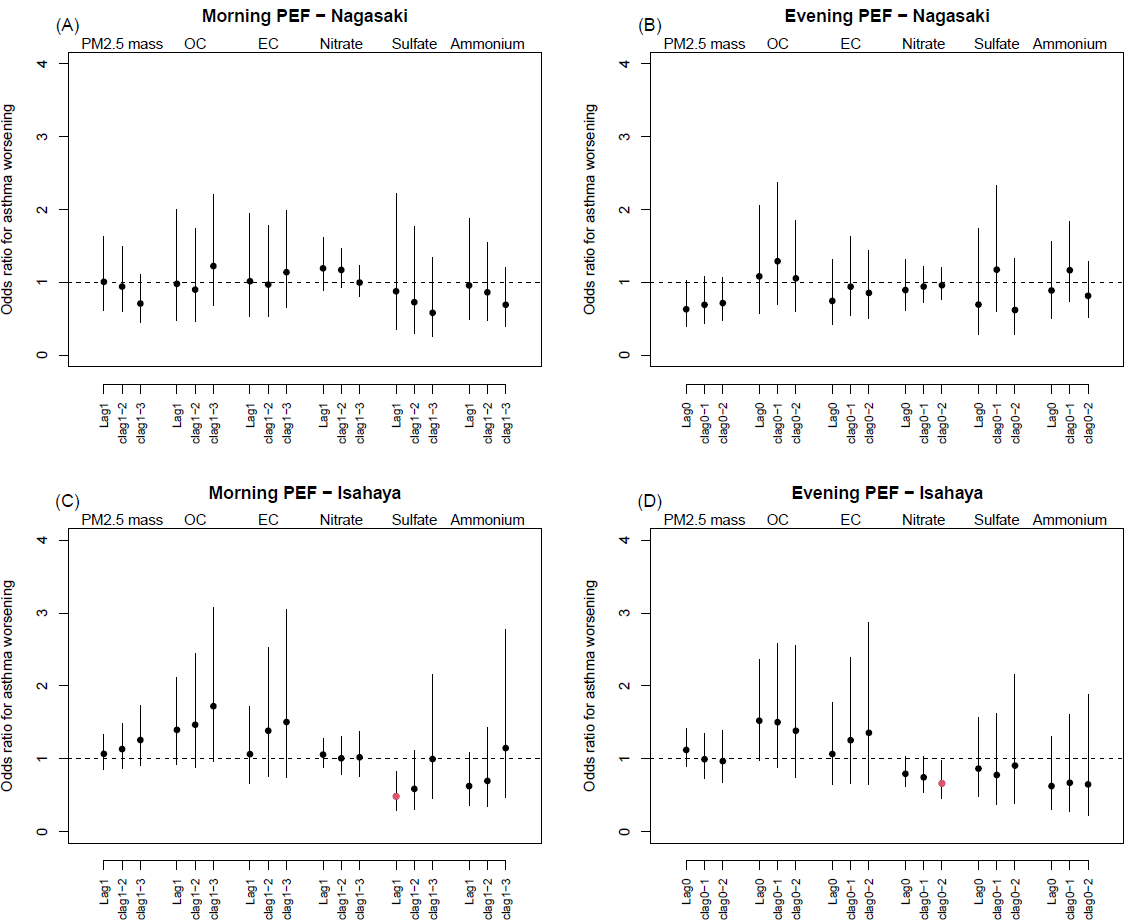


Figure S6. Odds ratios for the asthma worsening episodes, defined by the peak expiratory flow (PEF) reduction >20% from personal monthly maximum, per an interquartile range (IQR) increase in the PM_2.5_ exposures, estimated by the generalized linear mixed effect models adjusting for PM_2.5_ mass in Nagasaki city (A and B) and Isahaya city (C and D). Different lag days of the exposures were applied to the morning PEFs (A and C) on the preceding day (lag1), the two preceding days (clag1–2), and the three preceding days (clag1–3) and the evening PEF (B and D) on the current day (lag0), the current day and the preceding day (clag0–1), and up to two preceding days (clag0–2). OC: organic carbon; EC: elemental carbon.


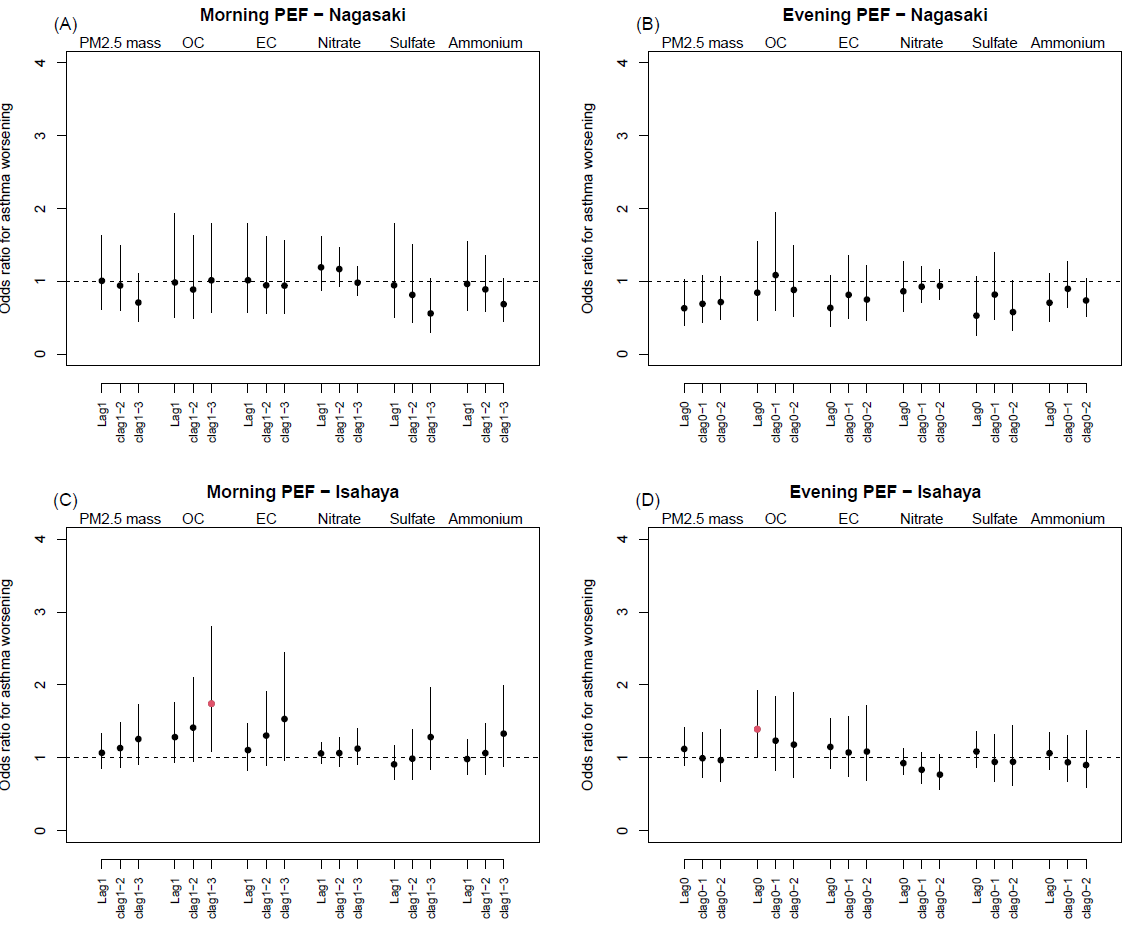


Figure S7. Odds ratios for the asthma worsening episodes, defined by the peak expiratory flow (PEF) reduction >20% from personal monthly maximum, per an interquartile range (IQR) increase in the PM_2.5_ exposures, estimated by the generalized linear mixed effect models with no adjustment of PM_2.5_ mass in Nagasaki city (A and B) and Isahaya city (C and D). Different lag days of the exposures were applied to the morning PEFs (A and C) on the preceding day (lag1), the two preceding days (clag1–2), and the three preceding days (clag1–3) and the evening PEF (B and D) on the current day (lag0), the current day and the preceding day (clag0–1), and up to two preceding days (clag0–2). OC: organic carbon; EC: elemental carbon.


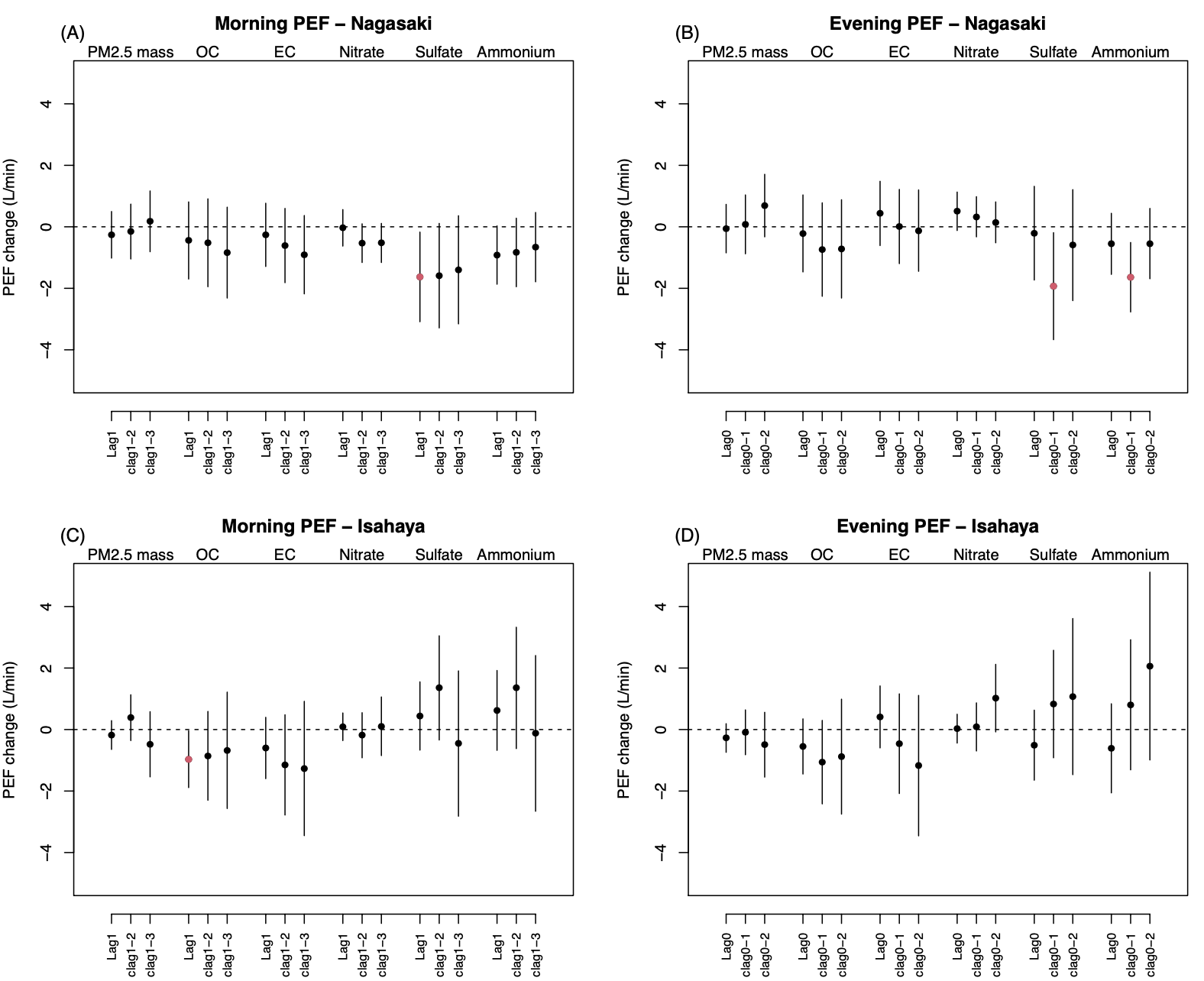


Figure S8. Results of sensitivity analysis with additional adjustment of medication use for asthma. Changes in the daily peak expiratory flow (PEF) per an interquartile range (IQR) increase in concentrations of PM_2.5_ total mass and five chemical species in Nagasaki city (A and B) and Isahaya city (C and D), estimated by the linear mixed effects model adjusting for potential confounders and PM_2.5_ mass. Different lag days were applied to the morning PEFs (A and C) on the preceding day (lag1), the cumulative exposure during two preceding days (clag1–2), and the cumulative exposure during three preceding days (clag1–3) and the evening PEF (B and D) on the current day (lag0), the cumulative exposure on the current day and the preceding day (clag0–1), and the cumulative exposure up to two preceding days (clag0–2). OC: organic carbon; EC: elemental carbon.


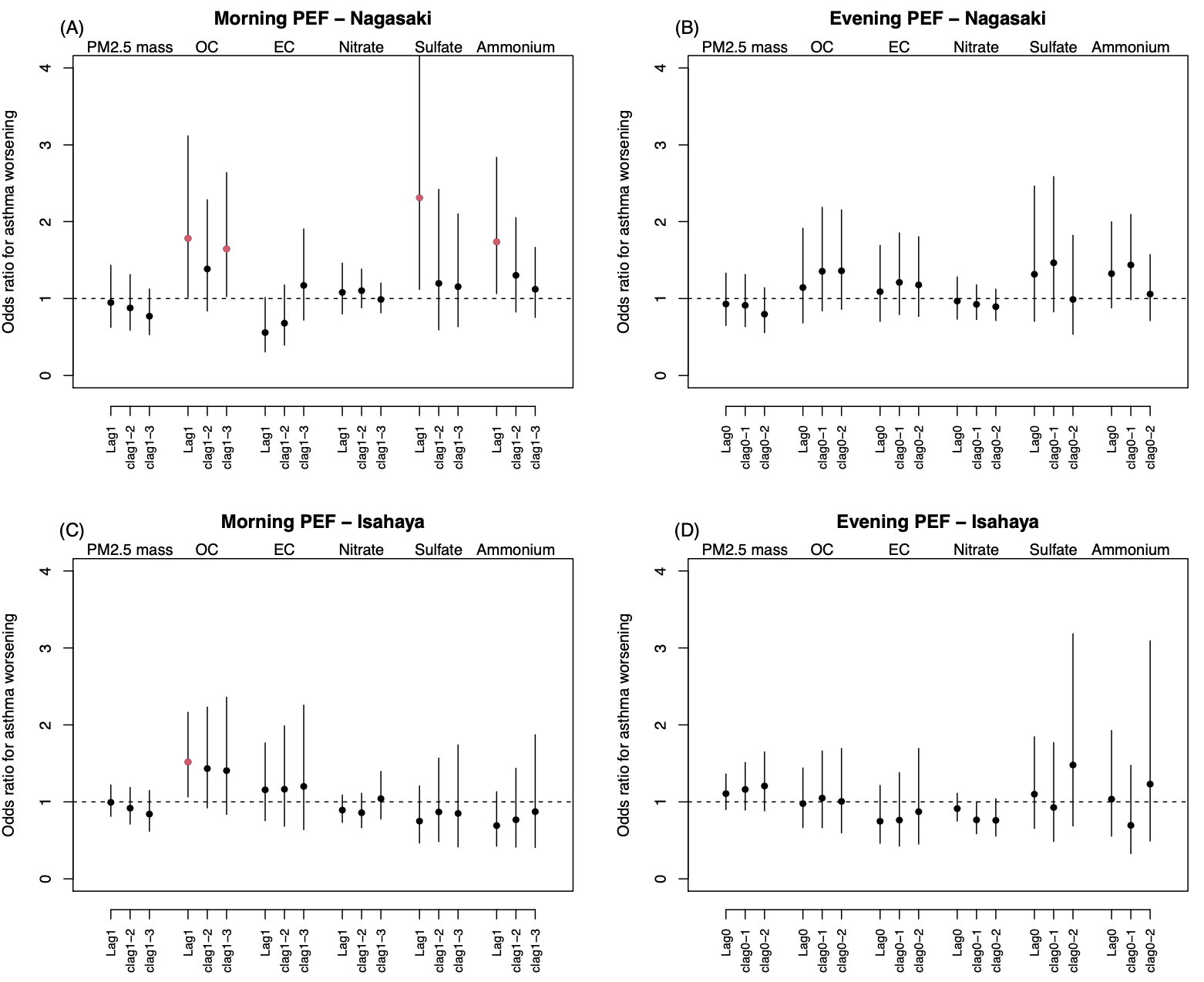


Figure S9. Results of sensitivity analysis with additional adjustment of medication use for asthma. Odds ratios for the asthma worsening episodes per an interquartile range (IQR) increase in concentrations of PM_2.5_ total mass and five chemical species in Nagasaki city (A and B) and Isahaya city (C and D), estimated by the generalized linear mixed effects model adjusting for potential confounders and PM_2.5_ mass. The episode was defined if the percent reduction in daily peak expiratory flow (PEF) from the personal best monthly PEF was >15% within a rolling 1-week window. Different lag days were applied to the morning PEFs (A and C) on the preceding day (lag1), the cumulative exposure during two preceding days (clag1–2), and the cumulative exposure during three preceding days (clag1–3) and the evening PEF (B and D) on the current day (lag0), the cumulative exposure on the current day and the preceding day (clag0–1), and the cumulative exposure up to two preceding days (clag0–2). OC: organic carbon; EC: elemental carbon.


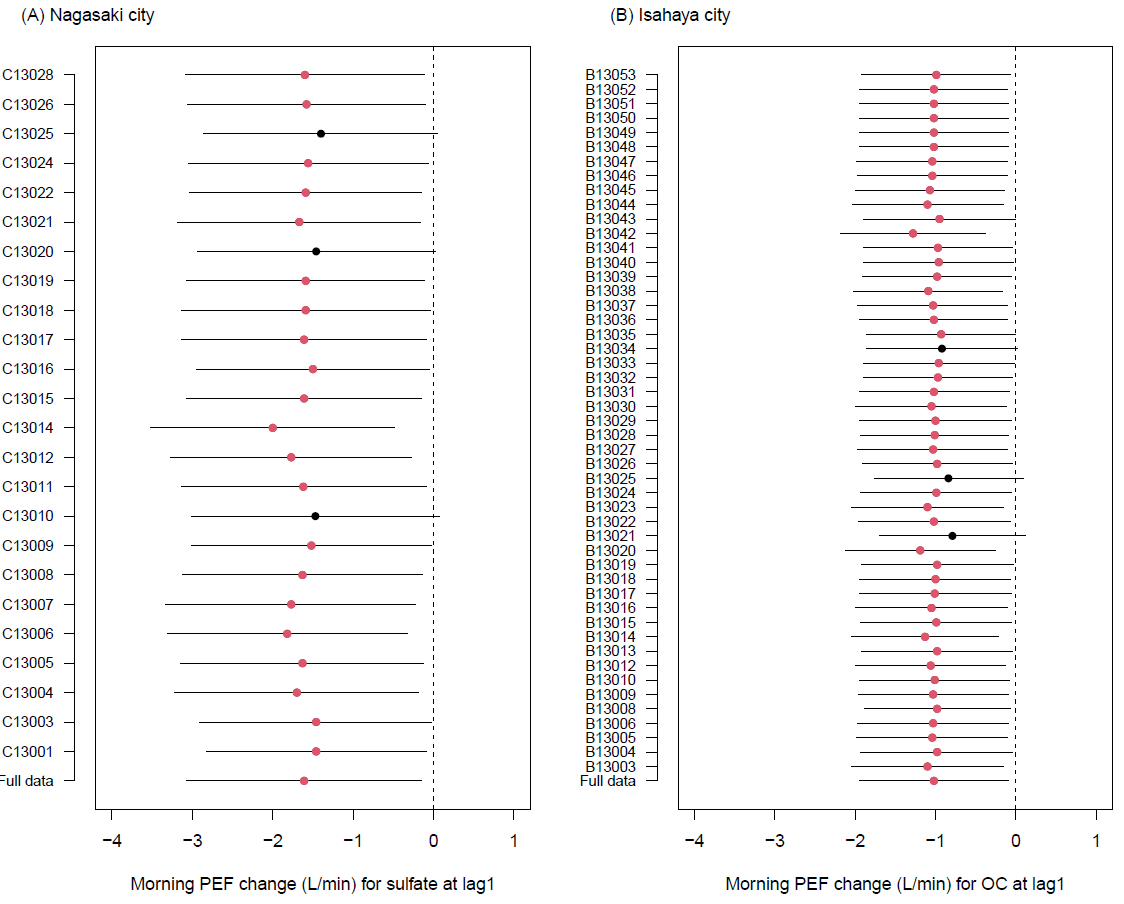


Figure S10. Results of the leave-one-out approach by children’s ID. The point estimates indicate the changes in the morning peak expiratory flow (PEF) per an interquartile range (IQR) increase in the concentration of sulfate at lag1in Nagasaki city (A) and OC at lag1 in Isahaya city (B), estimated by the linear mixed effect model adjusting for potential confounders and PM_2.5_ mass. The horizontal bars indicate the 95% confidence intervals. OC: organic carbon.
